# Developing an oncolytic Newcastle disease virus production process with EB66 cells

**DOI:** 10.1007/s00253-026-13889-9

**Published:** 2026-06-04

**Authors:** Lennart Jacobtorweihe, Annabelle Saulnier, Arnaud Léon, Yvonne Genzel, Udo Reichl

**Affiliations:** 1 Max Planck Institute for Dynamics of Complex Technical Systems, Magdeburg, Germany; 2 Valneva SE, Saint-Herblain, France; 3 Otto-Von-Guericke-University, Magdeburg, Germany

**Keywords:** Oncolytic virus production, Process development, Bioreactor, Semi-perfusion

## Abstract

Newcastle disease virus (NDV) has been the subject of extensive research as a potential oncolytic virus for various types of cancer. While clinical studies are ongoing, the manufacturing of high NDV doses on a large scale is challenging. It has previously been documented that Vero cells are capable of producing 2.4 × 10^8^ TCID_50_/mL in suspension batch production. However, the requirement of higher input doses and the challenging nature of establishing high cell density processes with Vero cells are significant obstacles in the effective utilization of these therapies. In pursuit of enhanced process intensification and the generation of viral vectors at elevated cell densities, EB66 cells have been identified as a highly effective producer cell line. In this study, the characteristics of EB66 cells in regard to NDV production were examined, with particular reference to cell growth and cell-specific virus productivity in batch and semi-perfusion mode. Optimal infection conditions for producing NDV in batch and semi-perfusion modes were identified for cultivation parameters including temperature, protease concentration (TrypLE), and multiplicity of infection. The favorable production conditions were then transferred to different batch processes using a stirred tank bioreactor and an orbital shaken bioreactor. These processes yielded up to 4.2 × 10^8^ TCID_50_/mL of the NDV LaSota strain with a cell-specific virus yield of 41 TCID_50_/cell. First semi-perfusion runs resulted in concentrations of 65 × 10^6^ cells/mL and an infectious virus titer of 7.5 × 10^8^ TCID_50_/mL. Finally, the potency of the produced viruses was evaluated, and a reduction in tumor size in mice after NDV injection was demonstrated. Overall, these results indicate that EB66 cells could be a viable host for producing oncolytic NDV.

## Introduction

Oncolytic viruses can selectively lyse cancer cells, while healthy cells with intact intracellular defense mechanisms, such as an interferon response, can interrupt virus replication (Chiocca 2002; Vaha-Koskela et al. 2007). In recent decades, there has been a notable increase in scientific research and clinical trials involving oncolytic viruses. A significant milestone was the approval of Talimogen Laherparepvec (T-VEC), an oncolytic Herpes simplex-1 (HSV-1) virus-based therapeutic against skin cancer, by the EMA and FDA (Lawler et al. 2017; Shalhout 2023). This has initiated a rapid development of a range of virotherapies. However, the production and manufacturing of many of these oncolytic viruses remains challenging. In the case of T-VEC, the recommended dosage is 10^6^ PFU/mL for the initial application and 10^8 ^PFU/mL for subsequent doses, with a maximum volume of 4 mL per dose (European-Medicines-Agency n.d; Lawler et al. 2017). Other therapies may require higher input doses with > 10^10 ^infectious viruses (Chaurasiya et al. 2021). Production processes have to be highly optimized, by identifying suitable production cell lines and optimal production conditions that comply with the relevant regulatory and GMP standards.

In addition to HSV-1, a number of oncolytic viruses have been documented, including VSV (vesicular stomatitis virus), MV (measles virus), and NDV (Newcastle disease virus) (Lawler et al. 2017; Sinkovics And Horvath 2000). The oncolytic activity of NDV in newborn mice was first discovered during the 1950 s (Sinkovics 1957). Since the 1990 s, more than 10 clinical studies with NDV as therapeutic agent for multiple types of cancer, such as melanoma, colorectal cancer, glioblastoma, or breast cancer, have been conducted. These studies have demonstrated the agent’s remarkable safety profile with no occurrence of severe adverse events, and a notable enhancement in overall survival rates (Schirrmacher 2016; Yang 2024). Production of the lentogenic NDV LaSota strain as oncolytic agent and veterinary vaccine mainly relies on eggs. However, production in embryonated chicken eggs brings several limitations, such as difficulties in scalability, lower efficiency, egg-to-egg variances, contaminated waste, supply chain difficulties, and further concerns (Hegde 2015; Zinnecker et al. 2024). Cell culture-based processes for NDV are reported by Jug et al. and Fulber et al. using either quail suspension cells (CCX.E10) or suspension Vero cells in batch mode. The maximum infectious virus titer that was obtained was 9 log_10_ FFU units/mL NDV LaSota and 2.37 × 10^8^ TCID_50_/mL NDV LaSota GFP (Fulber 2021; Jug et al. 2013).

Depending on the virulence of the strain, NDV can cause severe illness and death in poultry as it is an avian pathogen that is apathogenic in human. NDV is an enveloped virus with a size of approximately 100 to 200 nm and six different proteins (F, HA, N, M, L, P) are coded by a negative sense single mRNA. The sequence for protein P can be alternatively translated to V and W proteins (Burman et al. 2020; Sinkovics And Horvath 2000). The V protein plays an essential role in infection of avian cells since it is an antagonist to IFN I and hence the cell inner response to pathogens can be inhibited. However V is only an antagonist for avian IFN I and not for human IFN I. Cancer cells have a defect in their interferon pathway to enable unlimited growth; this also affects cell responses to pathogens. Reichard et al. reported a 10.000-fold higher replication rate of NDV in cancer cells compared to normal human cells for example (Reichard 1992). Mesogenic and velogenic strains demonstrate the highest degree of pathogenicity in avian species and are consequently seldom employed in clinical studies. Therefore, lentogenic strains such as the LaSota strain are preferred. However, for infection, the fusion proteins of these lentogenic strains require cleavage (Burman et al. 2020; Dortmans 2011). Consequently, protease addition (e.g., TrypLE) is necessary in the NDV production processes (Fulber And Kamen 2022). Out of all these variations, oncolytic activity between strains can differ strongly (Sinkovics And Horvath 2000).

In this study, the production of an oncolytic LaSota NDV strain is evaluated in avian EB66 suspension cells (Valneva SE). EB66 cells were derived from embryonic stem cells of Peking duck and are free of endogenous retroviruses (Genzel 2015; Guehenneux 2007; Olivier 2010). With short doubling times of 12 to 15 h, EB66 cells are able to reach high cell densities of up to 1.6 × 10^8^cells/mL in perfusion mode (Nikolay 2018; Olivier 2010). Moreover, EB66 cells can be seeded at relatively low cell concentrations (Madeline et al. 2015). Compared to other continuous cell lines, EB66 cells consume only low amounts of glutamine and tend to accumulate only little lactate and ammonium (Olivier 2010). This metabolic profile is a clear advantage, since accumulation of 2–3 mM ammonium and 20–30 mM lactate can already have a negative impact on cell growth, viability, or productivity (Schneider et al. 1996). EB66 cells are currently used for the production of nine approved vaccines (human (pandemic influenza) and veterinary (e.g., avian influenza, parvovirus, infectious bursal disease virus)) (Valneva 2026). In addition, academic studies reported the production of yellow fever virus and Zika virus in EB66 cells cultivated in stirred tank reactors (STR) with infectious titers of 7.3 × 10^8^ PFU/mL and 1.0 × 10^10^PFU/mL, respectively (Nikolay 2018). Moreover, EB66 cell growth has been demonstrated in scales up to 4500 L STRs (Valneva 2026).

Here, we present an evaluation of relevant cultivation parameters for the production of NDV in EB66 cells, such as multiplicity of infection (MOI), TrypLE activity, and temperature, at low and high cell concentrations for batch and semi-perfusion cultivation, as well as the transfer of production to a 3 L STR and 3 L orbital shaken bioreactor (OSB). Moreover, the produced material was evaluated for its potency compared to traditional egg production.

## Materials and methods

### Cell culture, media, and virus seed

EB66 cells were cultured in CDM4 Avian medium (Cytiva) with 2.5 mM glutamine (Merck) in non-baffled shake flasks (125 mL, working volume (wv) of 50 mL) at 37 °C, 150 rpm (50 mm throw), and 7.5% CO_2_. Passages were performed three times per week seeding at 0.3 × 10^6^ cells/mL. For the determination of infectious virus titer, adherent Vero cells (ECACC 88020401) were cultured in GMEM (Thermo Fisher Scientific) with 10% FCS (Fetal calf serum, Thermo Fisher Scientific) at 37 °C and 5% CO_2_, with routine passages twice per week. HepG2 (ATCC HB-8065) cells were maintained in MEM with EBSS NEAA NaPyr (Lonza) supplemented with 10% FCS and 2 mM L-glutamine at 5% CO_2_ and 37 °C. A549 (ECACC 86012804) and Hela (ATCC CCL-2) cells were cultured in DMEM (Lonza) supplemented with 10% FBS and 2 mM L-glutamine at 5% CO_2_ and 37 °C.

The lentogenic NDV LaSota strain used for all experiments was generated by reverse genetics. DNA fragments encoding the entire NDV genome (Genbank AF077761), as well as a GFP-sequence, were inserted into a pBR322 plasmid enriched with additional restriction enzyme sites (pLaSota). Viruses were rescued from a co-transfection of the pLaSota expression vector, and helper plasmids encoding for NDV NP, P, L and the T7 polymerase in EB66 cells (Peeters 1999; Romer-Oberdorfer 1999). The rescued viral particles were further propagated in either EB66, Hela cells, or chicken eggs and purified using a 20% sucrose cushion ultracentrifugation. Infection studies were performed using NDV at a MOI of 10^−4^. Master and working virus seed was generated in EB66 cells, and taken from the supernatant after centrifugation at 3000 × g for 5 min (titer of 3.0 × 10^8^ TCID_50_/mL).

### Batch cultivation in shake flasks

Batch cultures (50 mL wv) were seeded at 0.3 × 10^6^ cells/mL in 125-mL shake flasks under the same conditions as for routine passaging. Infections were performed when cell concentrations exceeded 5.0 × 10^6^ cells/mL. For infection, 20 mL of the culture was infected (rest was discarded) at a MOI of 10^−4^ with NDV working virus seed and temperature was set to 33 °C. After 1-h incubation at reduced working volume to increase the chance of virus-cell contact, 30-mL production medium containing higher levels of glutamine (4 mM), glucose (5 g/L), and TrypLE for an overall concentration of 2.5 U/mL was added. This resulted in a reference infection run with infection of 2.0 × 10^6^ cells/mL at MOI of 10^−4^.

### Semi-perfusion cultivations in shake flasks

EB66 cells were cultured according to a cell-specific perfusion rate (CSPR) of 36 pL/(cell × day). CSPR was calculated according to literature, based on glucose uptake and cell-specific growth rate (Bissinger 2019; Vazquez-Ramirez 2018). Therefore, viable cell concentration (VCC) was measured every 24 to 8 h and medium was manually replaced according to the CSPR. Cells were transferred to 50-mL spin tubes and centrifuged at 300 × g for 5 min. When the exchange volume exceeded 45 mL, the time between media exchanges was shortened to every 12 h or below.

### Cultivations in bioreactors

A DasGip System (1 L vessel, Eppendorf) was used for batch cultivations with a starting volume of 300 mL and a final wv of 750 mL post-infection. EB66 cells were stirred at 150 rpm using a pitched blade impeller. The pH value was controlled to 7.2 using base (NaHCO_3_) and CO_2_ via headspace gassing with a dead band of 0.05. A L-drilled hole sparger with air flow rates ranging from 3 to 9 L/h air was used to maintain a dissolved oxygen concentration (DO) above 50%.

An OSB (3 L single-use bag) with a wv of 1.5 L (SB10-X with 3 L adapter, Adolf Kühner AG) was used and operated at 90 rpm (transfer according to the Kühner manual and previous experiments performed using other cell lines (Göbel 2023)). Here, oxygen was provided via head space aeration with an air overlay at 300 mL/min resulting in a DO of above 50%.

At time of infection, virus seed was added, temperature was set to 33 °C, and the stirrer speed in the STR reduced to 100 rpm. One hour post-infection, 1.5-fold volume of fresh medium was added and the stirrer speed set to the previous value. Samples were taken every 24 h pre-infection and every 12 h post-infection to determine the external pH value, osmolality, VCC, metabolites, and TCID_50_ (for samples post-infection).

### Cell proliferation assay

A cytotoxicity 3–4,5-dimethyl-thiazolyl-2,5-diphenyltetrazolium bromide (MTT) assay was used to quantify cell proliferation after exposure to the NDV virus. For A549, Hela and HepG2 cells, 1, 4, and 5 × 10^4^ cells per well were seeded in 96-well plates, respectively, and allowed to attach for 24 h. Subsequently, NDV at various MOI or Mock (no virus addition) as a control were applied in medium supplemented with 1% FCS. The NDV samples were purified using a 20% sucrose cushion ultracentrifugation. After 96 h of incubation at 37 °C and 5% CO_2_, 5 µL of a Triton X-100 solution (2%) was added to wells reserved as cell killing control and 20 μL of MTT solution (5 mg/mL) was added to all wells. Four hours later, the solution of the wells was discarded, and 100 μL of dimethyl sulfoxide (DMSO) was added. The absorption at 570 nm (OD570) was measured on a microplate reader.

### In vivo* study*

For the study, the welfare of the animals was maintained in accordance with the general principles governing the use of animals in experiments of the European Communities and German legislation. The study was done in accordance with the United Kingdom Coordinating Committee on Cancer Research regulations for the Welfare of Animals (Workman 2010) and of the German Animal Protection Law and approved by the local responsible authorities, Berlin, Germany (Approval No. Reg 0010/19, LaGeSo Berlin, Germany).

Groups of seven female C57/Bl6 mice were injected intradermally with 4 × 10^5^ B16-F10 (NCI, USA) cells in their flank on day 0. On days 3, 5, 7, and 9, mice were treated with 60-µL intratumoral injections of NDV in PBS (1 × 10^7.5^ TCID_50_). The injected NDV was purified using a 20% sucrose cushion ultracentrifugation. The control group received an intratumoral injection of PBS. Tumor size was monitored during 49 days by measurement with a caliper. Tumor volume was estimated using the following formula: length × width × height/2.

### Other analytics and calculations

For the determination of VCC and viability, a ViCell XR by Beckman Coulter was used. Determination of metabolites (glucose, glutamine, glutamate, lactate, ammonia concentrations) was performed with the Cedex (Roche). Therefore, samples were centrifuged at 3000 × g for 5 min and supernatant was measured with the Cedex. Virus containing samples were previously heat inactivated at 80 °C for 5 min.

Infectious virus titers were measured using the tissue culture infectious dose 50 (TCID_50_) assay. Therefore, 96-well plates were seeded with adherent Vero E6 cells at 0.5 × 10^6^ cells/mL in GMEM + 5% FCS 1 day before infection. Virus samples were diluted in tenfold dilution rows containing GMEM, 1% gentamicin, and 5 U/mL porcine trypsin (Thermo Fisher Scientific). At the day of infection, prepared 96-well plates were washed twice with 1 × PBS and dilutions were transferred to the plates in eightfold replicates. Four days post-infection, positive wells (GFP-positive wells) were counted via fluorescence microscopy and infectious virus titers were calculated according to Spearman and Kärber. This TCID_50_ assay has an error of ± 0.3 log_10_. For the evaluation of the results, the maximum cell-specific growth rate *µ*_max_, CSVY, and volumetric virus productivity (VVP) were calculated as reported before (Jacobtorweihe 2025).

1 $${\mu}_{{m}{a}{x}}=\frac{{l}{n}({X}_{t2}/{X}_{t1})}{({t}_{2}-{t}_{1})}$$

*µ*_max_ is the maximum cell-specific growth rate (h^−1^) with *X*_*t*1_ (h), *X*_*t*2_ (h) based on the VCC at the beginning (*t*1) and end (*t*2) of the exponential growth phase. Reported values for *µ*_max_ post-infection refer to the same equation. The CSVY [TCID_50_/cell] was calculated as follows:

2 $${C}{S}{V}{Y}=\frac{{c}_{{v}{i}{r},{m}{a}{x}}-{c}_{{v}{i}{r}, {i}{n}{f}}}{{X}_{{m}{a}{x}}}$$

where *c*_vir,max_ is the maximum infectious virus concentration (TCID_50_/mL), *C*_vir,inf_ is the virus concentration added at time of infection (TCID_50_/mL), and *X*_max_ is the maximum VCC post-infection (cells/mL). The VVP [TCID_50_/(mL × h)] was determined as follows:

3 $${V}{V}{P}=\frac{\sum_{{t}_{0}}^{{t}_{n}}({{T}{C}{I}{D}}_{50 H,{t}_{n}}{\times}_{H, {t}_{n}})}{\sum_{{t}_{0}}^{{t}_{n}}({V}_{H, {t}_{n}}){\times t}_{n}}$$

where TCID_50 *H*,*tn*_ is the infectious virus titer of the harvest (*H*) at time *n* (TCID_50_/mL) and *V*_*H*,*tn*_ is the harvest volume at time *n* (mL).

### Statistical analysis

Mann and Whitney test was used to identify, whether the data measured during the oncolytic potency testing had the same distribution regarding the origin of the sample. Calculations were made using GraphPad PRISM.

## Results

In this study, we evaluated the production of an oncolytic NDV using EB66 cells on a small scale. We focused on cell growth characteristics and cell viability, optimal infection conditions in shake flasks, virus yields in bioreactors, and the potency of NDV produced in various hosts in order to identify optimal culture conditions.

### EB66 cell growth characteristics

First, we investigated the cell growth characteristics of EB66 cells and benchmarked the results against previous results from our group and an industry partner (Valneva). The obtained values, such as doubling times, specific cell growth rates, metabolite profiles, and maximal VCC in batch mode, should help to set feeding regimes towards cultivation at higher cell densities in semi-perfusion and perfusion.

**Fig. 1:**
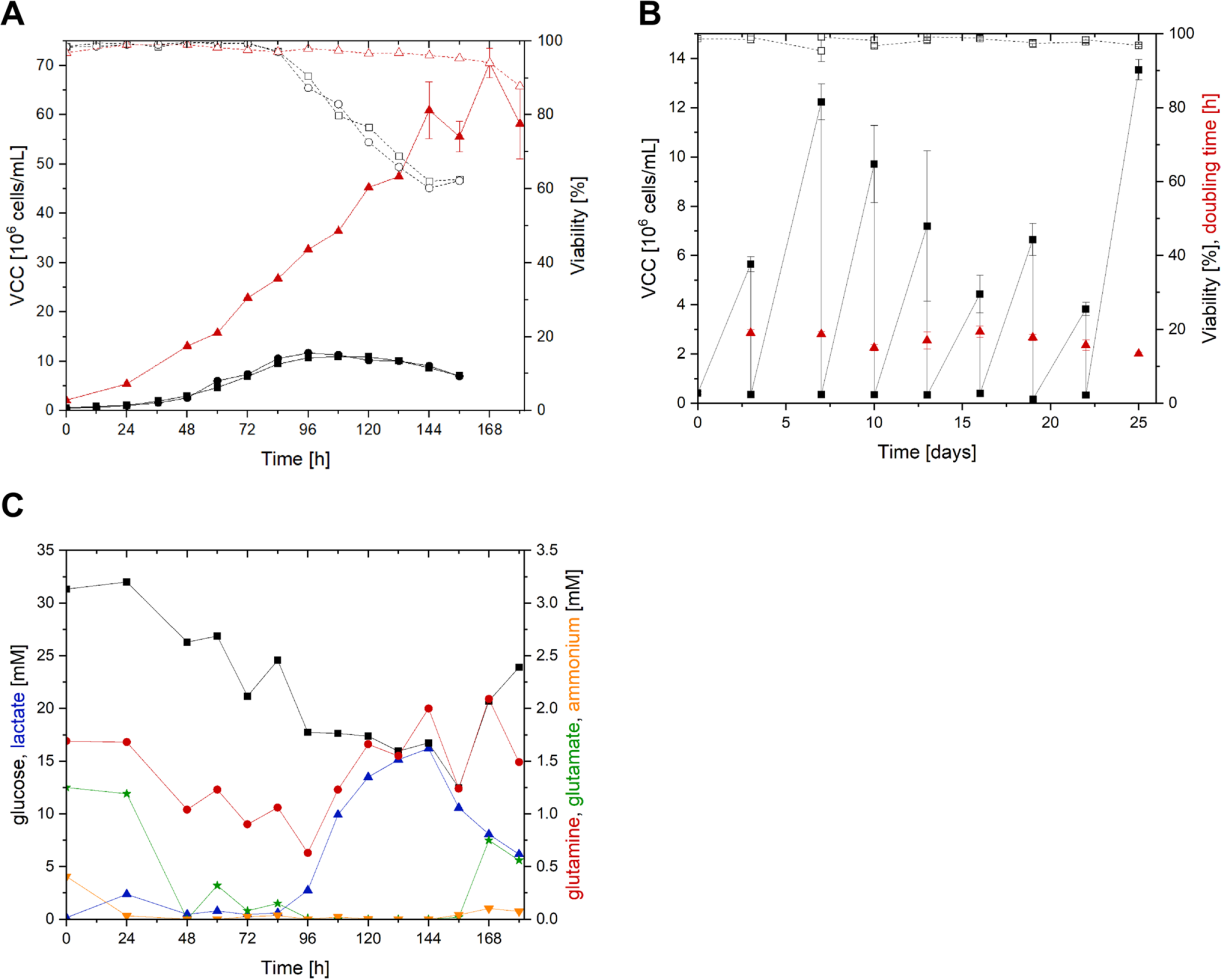
**A** Growth of EB66 cells in shake flasks in batch mode (●/∎) or semi-perfusion (  ) with VCC (full symbols) and viability (empty symbols). Semi-perfusion was started after 48 h and set to 1.6 reactor volumes per day (RV/day). Error bars refer to the mean and standard deviation of triplicate measurements. **B** VCC (full symbols) and viability (empty symbols) during routine passage of the EB66 cell culture, with the corresponding doubling times (  ). Error bars refer to mean and standard deviation of biological triplicates. **C **Metabolite concentrations of EB66 cells during the semi-perfusion cultivation shown in **A**

After inoculation at 0.3 × 10^6^ cells/mL, EB66 cells routinely reached cell concentrations of 6.0 × 10^6^ cells/mL within 3 days, equaling doubling times of 15.0 h. When cultured in batch mode, the cells reached a maximum concentration of 12.0 × 10^6^ cells/mL after 4 days with a *µ*_max_ of 0.036 h^−1^ (Fig. 1B). Viabilities stayed above 95% up to 84 h post-inoculation. However, until the end of batch cultivation at 156 h, viabilities constantly decreased to 60%. When cultured in semi-perfusion mode, EB66 cells peaked at 65.0 × 10^6^ cells/mL. Compared to the batch process, *µ*_max_ was decreased by 16% reaching 0.030 h^−1^ and a doubling time of 23.1 h. During semi-perfusion, cell growth was not limited by glucose concentration (Fig. 1C).

As expected for EB66 cells, glutamine consumption and ammonia release were low. Lactate concentration increased starting at 84 h, reaching 17 mM at 144 h. Until the end of the cultivation (187 h), lactate levels decreased, while glucose levels increased indicating a lower nutrient uptake in relation to the perfusion rate, caused by stagnation of cell growth and cell death. In semi-perfusion mode, at cell concentrations above 50.0 × 10^6^ cells/mL, EB66 cells tended to form aggregates, even with a TrypLE concentration of 1.5 U/mL, which impacted the accuracy of the cell count measurements (larger error bars). Viability remained above 90% until nearly the end of the semi-perfusion cultivation.

### Scouting for optimal infection conditions on a small scale in shake flasks

Several production parameters are considered important for virus production according to literature (Pelz 2022). Reduction of the process temperature, as it is done for influenza A virus (IAV), as well as the impact of MOI and the addition of TrypLE for activation of the NDV fusion protein, were tested in shake flask experiments.

**Fig. 2:**
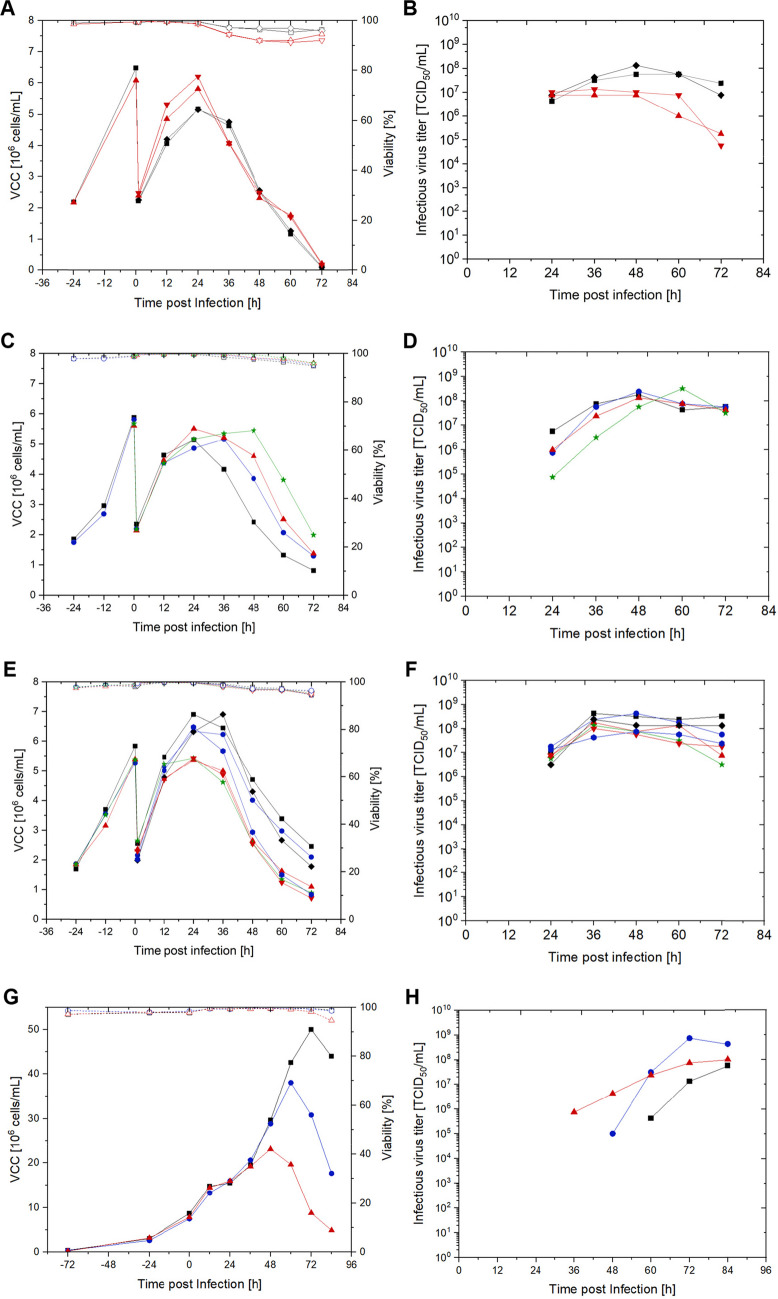
Scouting experiments to determine optimal infection conditions for NDV production in EB66 cells in shake flasks. **A**, **B** Variation of temperature with 33 °C (■/♦) and 37 °C (  /  ) post-infection at MOI of 10^−4^ and 5.0 U/mL TrypLE. **A** VCC and viability, **B** infectious virus titer. **C**, **D** MOI in batch mode: MOI 10^−2^ (■), MOI 10^−3^ (  ), MOI 10^−4^ (  ), MOI 10^−5^ (  ) at a TrypLE concentration of 5.0 U/mL TrypLE. **E**, **F** TrypLE activity in batch mode: 1.0 U/mL (■), 1.0 U/(mL × day) (♦), 2.5 U/mL (  ), 2.5 U/(mL × day) (  ), 5.0 U/mL (  ), 5.0 U/(mL × day) (  ), 7.5 U/(mL × day) (  ) at an MOI of 10^−4^. **G**, **H** MOI and TrypLE concentrations of MOI 10^−4^ and 1 U/mL TrypLE (■), MOI 10^−3^ and 2.5 U/mL TrypLE (  ), MOI 10^−4^ and 2.5 U/mL TrypLE (  ) with infection at 8.0 × 10.^6^ cells/mL in semi-perfusion mode. **G** VCC and viability, **H** infectious virus titer. VCCs are shown in full symbols, viability in empty symbols. Analytical error of the TCID_50_ assay equals ± 0.3 log_10_

Based on previous results from our project partners (Valneva SE), the impact of temperature reduction was tested at an MOI of 10^−4^ and 5.0 U/mL TrypLE (Fig. 2A and B). Next, scouting experiments were carried out to evaluate the impact of MOI and TrypLE activity. Here, ranges of MOI (10^−2^–10^−5^) and TrypLE (1.0–7.5 U/mL) were evaluated (Fig. 2C–F). To investigate the impact of MOI and TrypLE at high cell concentrations, the optimal MOI and TrypLE conditions were combined and tested at 8.0 × 10^6^ cells/mL (Fig. 2G and H).

To investigate the impact of a temperature shift on virus titer, EB66 cells were infected at either 33 °C or 37 °C in duplicates. Compared to the 37 °C culture, maximum VCC was 16% lower when cultured at 33 °C (Fig. 2A). The average difference in the virus titer was greater than or equal to one log from 48 h post-infection (hpi) onwards (Fig. 2B), with clearly higher cell viabilities and virus titers at 33 °C.

Testing different MOIs in batch mode cultivation with infection at 2.0 × 10^6^ cells/mL showed similar results for MOI conditions of 10^−2^ to 10^−4^ with maximum virus titers of 1.0–2.0 × 10^8^ TCID_50_/mL. For an MOI of 10^−5^, the highest virus titer was comparable, but reached peak titer only 12 h later. VCC post-infection decreased faster, the higher the MOI tested. Overall, the impact of varying MOI was lower relative to the impact of the temperature shift. MOI of 10^−3^ and 10^−4^ were chosen for a subsequent screening at higher cell concentrations. These conditions peaked their highest titer earlier than the MOI of 10^−5^ and would have a minor impact on virus seed costs as MOI 10^−2^ during potential scale-up.

For the addition of TrypLE, different strategies were tested. The addition of 5.0 U/mL, 7.5 U/mL and 5.0 U/(mL × day) in batch mode, led to the lowest maximal virus titers of 1–2 × 10^8^ TCID_50_/mL and VCCs with 5.3 × 10^6^ cells/mL (Fig. 2E). The widest difference of infectious virus titer was found at 72 h post-infection. Infections with TrypLE concentrations above 5.0 U/mL showed clearly lower infectious virus titer, resulting from a decrease of titer after reaching a maximum. Conditions of 1.0 U/mL, 2.5 U/mL, and 1.0 U/(mL × day) had the highest infectious virus titers with relatively constant values over time, reaching up to 4.5 × 10^8^ TCID_50_/mL. Overall, best conditions from this first scouting showed the highest virus production at 33 °C with an MOI of 10^−3^ and 1.0–2.5 U/mL TrypLE concentration.

When testing these conditions at higher cell concentrations in batch mode, a cell-density effect with lower productivity of the EB66 cells was observed (data not shown). Therefore, in a next step, variations of the best MOI and TrypLE concentrations in batch mode were tested in semi-perfusion mode to evaluate this cell-density effect further infecting at 10.0 × 10^6^ cells/mL (Fig. 2G and H). Faster virus production was observed at higher MOI and higher TrypLE conditions. The highest titer of 7.5 × 10^8^ TCID_50_/mL was measured at 2.5 U/mL TrypLE and MOI 10^−4^ with a CSVY of 20. These conditions were therefore chosen for further experiments. During the first 36 h of infection, no VCC difference between the tested conditions could be observed. Later time points then showed that at lower MOI and TrypLE concentration, peak VCC increased from 23.2 to 38.1 and 50.0 × 10^6^ cells/mL. The highest infectious virus titer did not correspond to the highest VCC. The highest titer of 7.5 × 10^8^ TCID_50_/mL was measured at 2.5 U/mL TrypLE and MOI 10^−4^ with a CSVY of 20. These conditions were therefore chosen for further experiments.

### Bioreactor cultivations

After scouting experiments in shake flasks, the production process was transferred to different bioreactor systems. An orbital shaken bioreactor (OSB) system, allowing to keep shaking conditions, was compared to a STR operated at different stirrer speeds and reactor volumes.

**Fig. 3:**
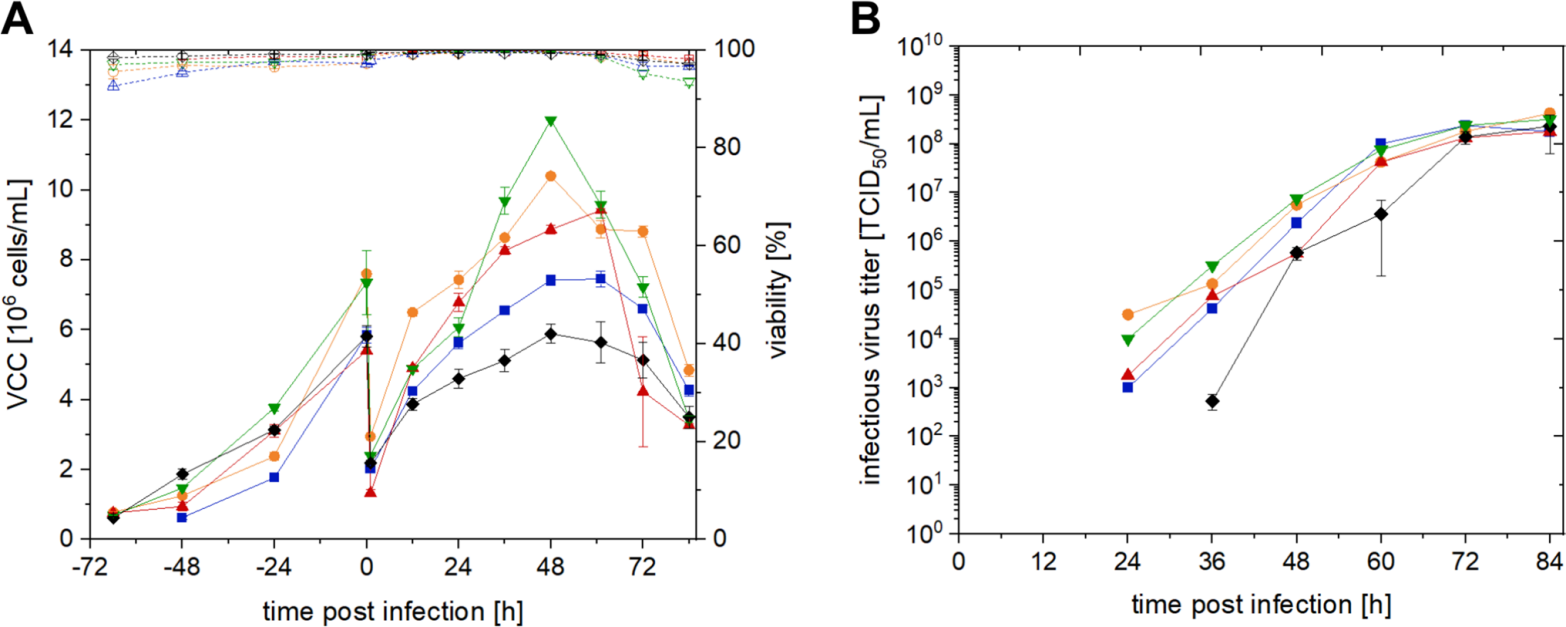
Comparison of different bioreactor systems and cultivation parameter settings for NDV production in EB66 cells. (  ) 1 L STR 150 rpm, (  ) 1 L STR 200 rpm, (  ) 3 L STR 150 rpm, (  ) 3 L OSB 3 L 90 rpm, (♦) 125 mL SF 125 mL 150 rpm **A** VCC (full symbols) and viability (empty symbols) for technical duplicates in STRs and OSB and biological triplicates in SF **B** infectious virus titer. Analytical error of the TCID_50_ assay equals ± 0.3 log_10_. For all runs, the temperature was decreased to 33 °C at TOI, infected with an MOI of 10^−4^ and diluted with fresh medium (2/5 cells and medium and 3/5 fresh medium, v/v). Error bars refer to mean and standard deviation

When stirrer speeds of 100 rpm and 250 rpm were tested, EB66 cells did not grow beyond 1.0 × 10^6^ cells/mL and exhibited decreased viability to values below 80% within 48 h (data not shown). Culturing and infecting EB66 cells at 150 rpm and 200 rpm resulted in similar maximum cell concentrations of 10.4 and 9.4 × 10^6^ cells/mL at 48 hpi and 60 hpi, respectively (Fig. 3A). At 200 rpm, a strong decrease in VCC of 65% was observed between 60 and 72 hpi, while at 150 rpm, a 45% decrease occurred 12 h later; viability remained high, probably due to the addition of TrypLE, which is necessary for viral infection but also lyses dead cells. Regarding infectious virus titer, both conditions peaked in a maximum at 84 hpi of 1.7 × 10^8^ (200 rpm) and 4.2 × 10^8^ TCID_50_/mL (150 rpm) (Fig. 3B). Referring to maximal titer and the CSVYs of 38 and 41, differences for both conditions were within the error of the TCID_50_-assay. Compared to the shake flask cultivations, cell concentrations post-infection were clearly higher in the bioreactor systems (up to 175% higher). When looking at the cell growth phase only, especially for the OSB cultivation, an improved cell growth rate could be seen (0.044 h^−1^) leading to higher maximal cell concentrations of 12.0 × 10^6^ cells/mL. This however resulted in a lower CSVY of 26 TCID_50_/cell, although maximal infectious virus titer was similar (STR = 3.2 × 10^8^ TCID_50_/mL, OSB = 4.2 × 10^8^ TCID_50_/mL). While up to 60 hpi infectious virus titer of all bioreactor conditions was clearly higher than in the shake flask, there was no difference in infectious virus titer at 72 and 84 hpi. Overall, it was possible to obtain similar values of infectious virus titer, CSVY, and VVP for all tested cultivation vessels (Table 1).

**Table 1:** Comparison of EB66 cell growth and NDV production in different cultivation vessels

Vessel Volume (L) Stirring/shaking (rpm)	**STR** 1 100	**STR** 1 150	**STR** 1 200	**STR** 1 250	**STR** 3 150	**OSB** 3 90	**SF (*n* = 3)** 0.05 150
***µ***_**max**_** (**h^−1^)		0.038	0.037		0.034	0.044	0.039
***T***_D_ (h)		18.2	18.7		20.4	15.6	17.8
VCC_max_ (10^6^ cells/mL)	1.0	10.4	9.4	1.0	7.5	12.0	5.9
Total process time (h)	48	144	144	48	132	144	144
Max. titer (10^8^ TCID_50_/mL)	-	4.2	1.8	-	2.4	3.2	2.3
CSVY (TCID_50_/cell)	-	41	19	-	32	26	39
VVP (10^6^ TCID_50_/(mL × h))	-	2.8	1.2	-	2.0	2.1	1.5

*STR* stirred tank reactor, *SF* shake flask, *OSB* orbital shaken bioreactor, *CSVY* cell-specific virus yield, *VCC*_*max*_ maximum viable cell concentration throughout the complete cultivation run, *VVP* volumetric virus productivity

### Potency

Taking into account FDA guidelines for viral products, potency, meaning the intended therapeutic effect of a drug, is a critical parameter (Food An Drug Administration 2011). As the host cell can have a major impact on potency of produced viruses, potency of NDV produced in EB66 cells was compared to material produced in HeLa cells or in embryonated chicken egg (Göbel 2022). NDV manufacturing traditionally relies on egg-based production (Santry 2018). The comparison to the egg-based produced NDV should demonstrate whether EB66 cells could be a promising host cell candidate. NDV oncolytic effect was tested in HeLa, HepG2, and A549 cells. All three cell lines are tumor cell lines originated from cancer tissue (cervical-, hepatocellular-, lung carcinoma), where NDV showed potential as oncolytic agent (An 2016; Yurchenko 2018).

Purified NDV material was applied at multiplicities of infection (MOI) of 10^−3^, 10^−1^, and 10. Phosphate-buffered saline (PBS) served as the negative control and Triton X-100 as the positive control, which caused 0% and 100% cytotoxicity in all cell lines, respectively.

**Fig. 4:**
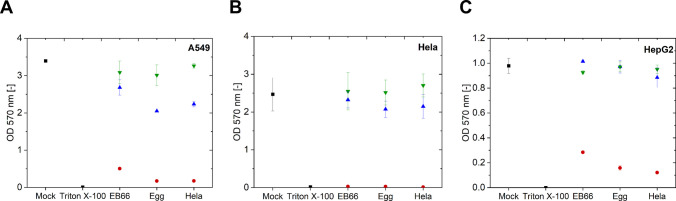
Results of a MTT cell viability assay to test the potency of NDV produced in EB66 cells, Hela cells, and embryonated chicken eggs. Optical density (OD) measurements of adherent cell cultures using **A** A549, **B** HeLa, and **C** HepG2 cells at 4 hpi at different MOIs (10  , 10^−1^  & 10^−3^  ) or controls ■ (PBS, Triton X-100). Error bars refer to mean of biological triplicates and standard deviation of the mean

When an MOI of 10^−3^ was applied, cell viability was indistinguishable from the PBS control for all three cell lines tested (Fig. 4A–C). No differences in production origin were observed. Testing at an MOI of 10^−1^ showed no effect on the viability of HeLa and HepG2 cells. However, in A549 cells, viability was reduced compared to the mock treatment. No production origin differences were observed in any of the three cell lines. At an MOI of 10, HeLa cells were fully lysed, with no differences observed between production origins. HepG2 cells exhibited low viability under all conditions. NDV originating from EB66 was 10% less toxic; however, this difference was not significant. A549 cells exhibited reduced viability with no observable difference regarding NDV origin. These results indicate a similar potency of NDV produced in EB66 cells compared to egg-based and Hela-based production, for all three tested cancer cell lines.

**Fig. 5:**
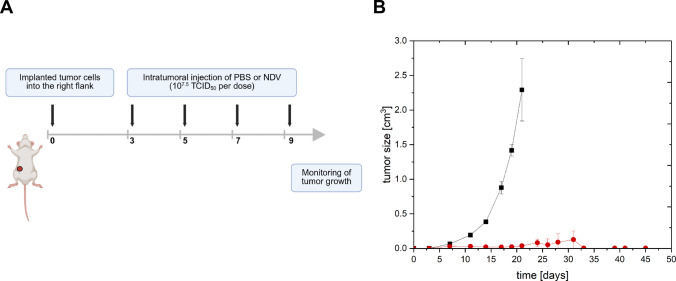
In vivo testing of NDV material produced in EB66 cells. **A** Seven mice per group (NDV and PBS as control) were injected after 3 days of tumor implantation, with a further injection at day 5, 7, and 9. Each NDV dose contained 10^7.5^ TCID_50_ (created with BioRender). **B** Mean tumor size of mice with NDV  and PBS ■ injection over 45 days. Error bars refer to mean of biological replicates (*n* = 7) and standard deviation of the mean

In a next step, the impact of a treatment of tumor cells implanted in mice with NDV produced in EB66 cells was tested by monitoring the tumor size over time. Mice were treated over 9 days with multiple NDV injections with doses of 10^7.5^ TCID_50_ given on days 3, 5, 7 and upon tumor implantation (*n* = 7) (Fig. 5A). An injection with PBS was performed as control (*n* = 7). Mean tumor size was then measured for 45 days. In the control group (PBS treated), five mice died between day 19 and day 21; no mice survived day 21. From the NDV-treated mice (*n* = 7), all mice survived until the end of the experiment (day 45). Average tumor sizes of up to 2.29 cm^3^ were measured in the control group. In contrast, the maximum average tumor size in the NDV-treated group was 0.13 cm^3^ on day 31; after that, no tumors were observed. The NDV-treated mice had significantly smaller tumors (day 19, *p* = 0.003) as the control group and had a longer overall survival (data not shown).

## Discussion

Previous studies on NDV production using Vero cells reported maximum virus titers of 2.0 × 10^8^ TCID_50_/mL (Fulber 2021). Assuming a high demand for the use of NDV in virotherapy and a need for high-titer doses, further process intensification to enable easy scaling-up and production at high cell concentrations (> 2.0 × 10^7^ cells/mL) is essential. However, suspension Vero cells are currently difficult to cultivate at concentrations above 6.0 × 10^6^cells/mL (Shen 2019). In contrast, EB66 cells exhibit rapid growth to high cell concentrations (> 1.6 × 10^8^cells/mL) and have demonstrated promising virus titers for various viruses including NDV (Leon 2016; Nikolay 2018; White 2018). Therefore, this study discusses an approach to characterizing and optimizing processes at a small scale, with the final goal to intensify these processes for large-scale manufacturing in bioreactors.

### EB66 growth characteristics

The observed cell growth and doubling times were consistent with those reported previously in batch cultivations of EB66 cells in two different media (Nikolay 2018). In those studies, doubling times of 17 h and 19 h with maximum VCCs of 2.0 × 10^7^ cells/mL and 1.4 × 10^7^cells/mL, respectively, were determined in GRO-I and CDM4 Avian medium. Compared to other avian suspension cells such as CCX.E10 (doubling time 26.6 h) or AGE.CR.pIX (30.0 h), EB66 cells exhibited rapid growth (15.0 h) (Göbel 2023; Lohr 2012). Previous work has reported a limitation of the maximum VCC to 2.5 × 10^7^ cells/m in semi-perfusion mode, with a ring of cells forming at the top of the flask. However, in perfusion mode using an ATF system (alternating tangential flow filtration) as a cell retention device, VCCs of 1.6 × 10^8^cells/mL were reached (Nikolay 2018). Batch cultivations confirmed the previously described values, whereas semi-perfusion mode resulted in VCCs of up to 6.5 × 10^7^cells/mL without the formation of a ring of cells. While cell growth in semi-perfusion was not limited by glucose concentration at the end of the semi-perfusion, periodic manual interventions by centrifugation and medium exchange can be a stress factor and might have limited the VCC here. In addition, other nutrients which were not measured in this study (e.g., vitamins or amino acids) might have been limiting in semi-perfusion mode. However, compared to the previous results, it was now possible to run small-scale experiments in parallel to optimize potential perfusion strategies (Nikolay 2018).

Moreover, EB66 cells are favorable for production at high cell density because they have a tendency to release only low levels of lactate and ammonium, both often described as growth and production limiting metabolites (Olivier 2010). During cultivation in semi-perfusion mode, moderate accumulation of lactate and increased glucose consumption (84 h onwards) were observed. This could indicate an insufficient perfusion rate. Indeed, the chosen volume exchange rate of 1.6 RV/day was lower than the CSPR used later. Consequently, the nutrient consumption of cells exceeded what was provided via the feed. Even higher cell concentrations could have been achieved using a CSPR based strategy. Overall, the cultivation of EB66 cells in both batch and semi-perfusion modes was successful, and the growth characteristics and metabolism were favorable for potential high cell density virus production.

### Scouting for optimal infection conditions on a small scale in shake flasks

The reported data on NDV production processes in adherent or suspension Vero cells describe a maximum VCC of 1.0–2.0 × 10^6^cells/mL, peaking at 24 hpi with an optimal infectious virus titer and a corresponding harvest time of 24 to 48 hpi (Arifin 2010; Fulber 2021). As a benchmark, maximum titers of 2.4 × 10^8^ TCID_50_/mL, corresponding to a CSVY of about 185 TCID_50_/cell, were reported using suspension Vero cells (Fulber 2021). For a quail suspension CCX.E10 cell line, a maximum titer of 9 log_10_FFU/mL was reported (Jug et al. 2013). For use as of NDV as an oncolytic virus, titers exceeding 1.0 × 10^9^ TCID_50_/mL seem to be required for a commercially viable manufacturing process.

Therefore, we evaluated NDV production in EB66 cells by varying the MOI, TrypLE activity, temperature, and VCC at TOI to determine the optimal conditions for virus production conditions. Here, even the initial, non-optimal evaluation yielded very promising titers of > 1.0 × 10^8^ TCID_50_/mL, which is close to the current benchmark for suspension Vero cells.

Reducing the process temperature has shown to have a beneficial effect in many virus production processes, including those involving HSV-1, IAV, and retroviruses (Lee 1998; Wechuck 2002; Wu 2021). A higher infectious virus titer may result from changes in cellular metabolism based on the temperature shift, as well as from higher virus stability at lower temperatures, e.g., due to decreased protease activity (Pelz 2022). Under the tested conditions, the NDV titer produced in EB66 cells was higher at 33 °C than at 37 °C. In contrast, Fulber et al. demonstrated for suspension Vero cells that a reduction of the process temperature had no beneficial effect (Fulber 2021). Similarly, a temperature shift did not improve infectious virus titer for production of YFV and ZIKV in EB66 cells (Nikolay 2018). Furthermore, we have observed a relatively short half-life time of 2.6 h at 37 °C and 4.1 h at 33 °C, respectively, for the tested NDV LaSota strain in cell culture supernatant (data not shown). This emphasizes the importance of production, harvesting, and storage strategies (Pelz 2022).

The production of NDV requires adding proteases to cleave the fusion protein and enable viral entry (Nagai et al. 1976). The activity of the added protease affects the overall infectious virus titer as well as cell concentrations and cell lysis; the latter two are highly relevant for downstream processing. Therefore, optimization for the respective medium, cultivation vessel, and culture mode is highly relevant. Typical added concentrations of proteases range from 1 to 5 µg/mL (≈10 to 50 U/mL), achieved through simple or multiple additions of trypsin or TrypLE. In this study, TrypLE addition resulted in high VCCs, likely because dead cells were rapidly degraded and subsequently undetectable via trypan blue measurement (Fulber 2021; Liu 2019; Nan 2021). The highest virus titer was measured at the lowest TrypLE concentration. Repeated TrypLE addition did not seem to be beneficial. Similar results were observed with trypsin addition for NDV production in Vero cells (Fulber And Kamen 2022).Nestler et al. showed that TrypLE lost only 15 % of its activity within eight days at 37 °C (Nestler 2004). Therefore, repeated addition does not seem relevant. Moreover, intensifying the process by increasing the cell concentration required further screening to determine the optimal TrypLE activity. Although the virus titer did not differ between 1 and 2.5 U/mL in batch mode, a tenfold higher titer was observed with 2.5 U/mL for high cell density cultures in semi-perfusion mode.

The MOI can significantly impact virus production in mammalian cell culture. In virological studies involving molecular or signaling mechanism, MOIs greater than 10 are often used. However, for manufacturing, the interest lies in producing viruses at the lowest possible MOI to allow for efficient scaling-up with the available seed virus (Pelz 2022). A higher MOI generally shortens the time needed to reach the peak virus titer, as demonstrated for rVSV-NDV in AGE1.CR.pIX and YF-ZIKprM/E in AGE.1CR cells (Göbel 2022, 2024). However, the use of a high MOI increases the risk of defective interfering particle accumulation. These particles replicate faster than wild-type virions due to defects in their genome, and can impact overall product yields (Frensing 2014). Finally, a higher MOI means higher production costs at larger scales. Therefore, the MOI is typically reduced to values between 10^−1^ and 10^−4^in many virus manufacturing processes (Pelz 2022). In this study, for batch and semi-perfusion runs, a MOI of 10^−4^resulted in best titers.Fulber et al. obtained similar values for NDV batch production using suspension Vero cells with infectious virus titers of about 1.0 × 10^8^ TCID_50_/mL within a MOI range of 10^−2^ to 10^−4^(Fulber 2021). Furthermore as reported byArifin et al., titers of up to 4.8 × 10^7^ TCID_50_/mL were reported for adherent Vero cells in microcarrier cultures at MOIs of 0.2 and 2 (Arifin 2010). Taken together, these observations indicate that a MOI of about 10^−4^ is the optimal compromise between productivity and cost.

### Bioreactor cultivations

When scaling up from shake flasks to bioreactor vessels, some process parameters must be reconsidered. For example, stirrer speed can significantly impact on cell growth and virus production due to its effect on mixing and shear stress. Low agitation speeds result in longer mixing times and may lead to limited nutrient or oxygen supply in certain zones of the bioreactor (Göbel 2023). Likewise the virus may not be homogeneously distributed for infection. Conversely too high of a stirring speed can slow down cell growth and decrease cell viability due to high shear force. However this may be beneficial for facilitating virus infection and virus release with respective cell stress and lysis.Göbel and Jaen evaluated stirring speeds of 80 130, and 180 rpm in a bioreactor setup similar to the one used in our study for producing oncolytic rVSV-NDV in quail-derived CCX.E10 cells. In that study, the infectious virus titer produced at 180 rpm was 32-fold times higher than at the lowest speed (Göbel 2023).

Surprisingly, EB66 cells did not grow when stirred at 100 rpm. However, other cells tested by our group in this setup, such as CCX.E10, MDCKsus, AGE.CR.pIX, or HEK293 cells, were successfully cultured at stirring speeds up to 100 rpm. Microscopy images taken during VCC measurements using a ViCell XR system showed strong aggregation of EB66 cells at this stirring speed. Generally, cell aggregation is often favorable for virus production due to facilitated cell-to-cell spreading of released viruses (Leon 2016; Li 2021). In this case, however, the too strong aggregation at a low stirring speed might have impacted VCC and viability. The experiment was repeated more than three times using different pre-cultures; however, there was no change in the outcome. Additionally, a stirring speed of 250 rpm equally impeded cell growth, possibly due to excessive shear force.

For the tested bioreactor setup, no difference in NDV production in EB66 cells was observed at stirrer speeds of 150 and 200 rpm. Therefore, the lower setting was preferred because a lower power input would reduce production costs in large-scale manufacturing and cause less shear stress. Cultivating EB66 cells alternatively in an OSB using standard settings found for other cell lines, such as AGE1.CR.pIX, was equally successful and even led to a slight increase in *µ*_max_and the peak VCC (Coronel 2019). The virus titer was slightly lower. This difference, however, seems to be within the margin of the error of the method and is therefore not significant. Further optimization of parameter settings could probably be beneficial for EB66 cell cultivation in an OSB. For producing of oncolytic rVSV-NDV in CCX.E10 cells in an OSB, reduced cell growth, a comparable titer, and a productivity similar to that of STR were observed (Göbel 2023). Within the scope of this study, we can conclude that OSB and STR cultivations resulted in similar results as experiments performed in shake flasks, providing a solid foundation for further scale-up and process development. To our knowledge, these are the first data on EB66 cell cultivation and virus production in an OSB system.

To meet the required input doses for oncolytic therapy and provide an affordable drug to patients, an infectious virus titer of 10^8^ TCID_50_/mL seems insufficient. Variations from the current batch processes that result in a titer greater than 10^8^ TCID_50_/mL must be addressed. Therefore, the potential limits of the batch process were evaluated to enable a more targeted optimization strategy. For producing viruses and viral vectors, such as IAV, ZIKV, YFV, or rVSV-NDV, increasing VCC at TOI is a common strategy for process intensification (Jacobtorweihe 2025; Nikolay 2020; Zinnecker 2025). However, a “cell-density effect,” which describes the reduction of CSVY at higher cell concentrations, interferes with this strategy (Kamen And Henry 2004). Fortunately, various setups using perfusion processes have demonstrated that this problem can be overcome. Recently, this was shown for oncolytic rVSV-NDV production achieving a threefold increase in infectious virus titer and a 1.9-fold higher VVP (Jacobtorweihe 2025). Testing elevated VCCs for NDV production in EB66 cells revealed that the addressed cell-density effect also restricts batch cultures. Moreover, our group closely investigated the thermostability of NDV LaSota (data not shown). We found a half-life of 4.1 h at 33 °C and 2.6 h at 37 °C in cell culture supernatant, meaning a substantial amount of the infectious viruses produced are probably degraded during the 36–84-h-long production process. Hence, continuously harvesting the produced viruses with cooling could be highly beneficial for further process improvement. For oncolytic NDV production, a full perfusion process with continuous virus harvesting seems necessary and will be investigated next.

### Potency

First, it was important to determine if EB66 cell-produced material could be used for virotherapies by measuring its oncolytic potential using an MTT assay, for example (Gujar 2024). In this case, the EB66 cell-produced material should demonstrate similar oncolytic potential to material produced in standard systems, such as embryonated chicken eggs or adherent HeLa cells. Additionally, an evaluation in mice should clarify whether EB66 cell-produced NDV could reduce tumor size while extending overall survival.

The oncolytic effect of a virus can differ greatly depending on cell lines used for testing. Because A549 and Hela cells are highly sensitive to NDV treatment, they were used in our study (Yurchenko 2018). The comparable potency of NDV produced in EB66 cells observed in this study is therefore an important step towards developing an alternative manufacturing method. At an MOI of 10, high cytotoxicity was observed in all three cell lines tested in the MTT assay. However, the dose-dependent effect observed in our results indicates the necessity of high input doses for successful treatment. A dose-dependent oncolytic effect was also observed with various other NDV strains tested on A549 and Hela cells (Pathak et al. 2022; Yurchenko 2018).

Moreover, the injection of EB66 cell produced NDV to treat tumor cells implanted in mice was highly efficient in vivo, resulting in complete tumor reduction and a 100% overall survival 45-day post-tumor implantation (compared to a 0% survival in the PBS control group). Similar results were obtained by injecting immunodeficient mice with 10^7^ TCID_50_oncolytic rVSV-NDV, which led to nearly double the overall survival in comparison to the PBS-treated mice (Abdullahi 2018). This experiment did not aim at determining the extent to which the reduction of tumor growth was mediated by direct oncolysis or immunostimulatory responses. Further studies on the induction (and quantification) of an immune response using NDV LaSota as oncolytic virus were conducted by Zamarin et al. (Zamarin 2017). Injections of NDV LaSota and the immune checkpoint protein CTLA-4 blockade (Cytotoxic T-Lymphocyte-Associated Protein 4) in B16-F10 mice led to reduced tumor size and measurable immune response not only at the intratumoral injection site, but also for distant tumors (Zamarin 2017). In summary, the initial experiments demonstrated that NDV produced using EB66 cells was comparable to that produced using standard methods. Moreover, EB66 cell-derived NDV had a potent effect in the murine model and prolonged overall survival.

## Summary

An initial evaluation of NDV production in suspension EB66 cells in batch mode is presented. The optimal infection conditions were determined to be a MOI of 10^−4^, with a single addition of 2.5 U/mL TrypLE at TOI and a temperature shift to 33 °C during the replication phase of the virus. These results were further confirmed through cultivations at higher cell densities in semi-perfusion mode. The EB66 cell-produced oncolytic NDV material retained its potency, and no significant differences in the oncolytic effect were observed compared to egg-based NDV material. The production process was successfully transferred to laboratory-scale STR and to an OSB. A peak infectious virus titer of 4.2 × 10^8^ TCID_50_/mL achieved in batch mode and 7.5 × 10^8^ TCID_50_/mL in semi-perfusion mode demonstrated that the EB66 cell line is a suitable host for oncolytic NDV production. The next step could be to develop a perfusion process with continuous virus harvesting to target titers exceeding 1 × 10^9^ TCID_50_/mL.

## Data Availability

Data are available in the article. Additional data are available upon reasonable request from the authors.

## Abbreviations

CSPR: Cell-specific perfusion rate
CSVY: Cell-specific virus yield
DO: Dissolved oxygen concentration
FCS: Fetal calf serum
GMO: Genetically modified organism
GMP: Good manufacturing practice
HCD: High cell density
IAV: Influenza A virus
MOI: Multiplicity of infection
NDV: Newcastle disease virus
OSB: Orbital shaken bioreactor
OV: Oncolytic virus
rpm: Rounds per minute
RV: Reactor volumes
STR: Stirred tank bioreactor
STY: Space time yield
TCID_50_: Tissue culture infectious dose 50
TOI: Time of infection
T-VEC: Talimogen Laherparepvec
VCC: Viable cell concentration
VSV: Vesicular stomatitis virus
VVP: Volumetric virus productivity
wv: Working volume
YFV: Yellow fever virus
ZIKV: Zika virus

## References

[CR1] Abdullahi S et al (2018) A novel chimeric oncolytic virus vector for improved safety and efficacy as a platform for the treatment of hepatocellular carcinoma. J Virol. 10.1128/JVI.01386-18 30232179 10.1128/JVI.01386-18 PMC6232488

[CR2] Administration, F.a.D (2011) Guidance for industry potency tests for cellular and gene therapy products. Available from: https://www.fda.gov/files/vaccines,%20blood%20%26%20biologics/published/Final-Guidance-for-Industry--Potency-Tests-for-Cellular-and-Gene-Therapy-Products.pdf

[CR3] An Y et al (2016) Recombinant Newcastle disease virus expressing P53 demonstrates promising antitumor efficiency in hepatoma model. J Biomed Sci 23(1):55 27465066 10.1186/s12929-016-0273-0 PMC4964062

[CR4] Arifin MA et al (2010) Production of Newcastle disease virus by Vero cells grown on cytodex 1 microcarriers in a 2-litre stirred tank bioreactor. J Biomed Biotechnol 2010:586363 20625497 10.1155/2010/586363 PMC2896699

[CR5] Bissinger T et al (2019) Semi-perfusion cultures of suspension MDCK cells enable high cell concentrations and efficient influenza A virus production. Vaccine 37(47):7003–7010 31047676 10.1016/j.vaccine.2019.04.054

[CR6] Burman B, Pesci G, Zamarin D (2020) Newcastle disease virus at the forefront of cancer immunotherapy. Cancers (Basel). 10.3390/cancers12123552 33260685 10.3390/cancers12123552 PMC7761210

[CR7] Chaurasiya S, Fong Y, Warner SG (2021) Oncolytic virotherapy for cancer: clinical experience. Biomedicines. 10.3390/biomedicines9040419 33924556 10.3390/biomedicines9040419 PMC8069290

[CR8] Chiocca EA (2002) Oncolytic viruses. Nat Rev Cancer 2(12):938–950 12459732 10.1038/nrc948

[CR9] Coronel J et al (2019) Influenza A virus production in a single-use orbital shaken bioreactor with ATF or TFF perfusion systems. Vaccine 37(47):7011–7018 31266669 10.1016/j.vaccine.2019.06.005

[CR10] Dortmans JC et al (2011) Virulence of Newcastle disease virus: what is known so far? Vet Res 42(1):122 22195547 10.1186/1297-9716-42-122 PMC3269386

[CR11] European-Medicines-Agency (n.d.) Imlygic, INN-talimogene laherparepvec. Available from: https://www.ema.europa.eu/en/documents/product-information/imlygic-epar-product-information_en.pdf#:~:text=Talimogene%20laherparepvec%20is%20an%20attenuated%20herpes%20simplex,in%20Vero%20cells%20by%20recombinant%20DNA%20technology. Accessed 22 Jan 2026

[CR12] Frensing T et al (2014) Impact of defective interfering particles on virus replication and antiviral host response in cell culture-based influenza vaccine production. Appl Microbiol Biotechnol 98(21):8999–9008 25132064 10.1007/s00253-014-5933-y

[CR13] Fulber JPC, Kamen AA (2022) Development and scalable production of Newcastle disease virus-vectored vaccines for human and veterinary use. Viruses. 10.3390/v14050975 35632717 10.3390/v14050975 PMC9143368

[CR14] Fulber JPC et al (2021) Process development for Newcastle disease virus-vectored vaccines in serum-free Vero cell suspension cultures. Vaccines. 10.3390/vaccines9111335 34835266 10.3390/vaccines9111335 PMC8623276

[CR15] Genzel Y (2015) Designing cell lines for viral vaccine production: where do we stand? Biotechnol J 10(5):728–740 25903999 10.1002/biot.201400388

[CR16] Göbel S et al (2022) Cell-line screening and process development for a fusogenic oncolytic virus in small-scale suspension cultures. Appl Microbiol Biotechnol 106(13–16):4945–4961 35767011 10.1007/s00253-022-12027-5 PMC9329169

[CR17] Göbel S et al (2023) Characterization of a quail suspension cell line for production of a fusogenic oncolytic virus. Biotechnol Bioeng. 10.1002/bit.28530 37584190 10.1002/bit.28530

[CR18] Göbel S et al (2024) Parallel multifactorial process optimization and intensification for high-yield production of live YF17D-vectored Zika vaccine. Vaccines. 10.3390/vaccines12070755 39066393 10.3390/vaccines12070755 PMC11281342

[CR19] Guehenneux F, Moreau K, Esnault M, Mehtali M, inventors; Vivalis SA, assignee (2010) Duck embryonic derived stem cell lines for the production of viral vaccines. United States patent application US 12/597,486

[CR20] Gujar S et al (2024) Tutorial: design, production and testing of oncolytic viruses for cancer immunotherapy. Nat Protoc 19(9):2540–2570 38769145 10.1038/s41596-024-00985-1

[CR21] Hegde NR (2015) Cell culture-based influenza vaccines: a necessary and indispensable investment for the future. Hum Vaccin Immunother 11(5):1223–1234 25875691 10.1080/21645515.2015.1016666 PMC4514150

[CR22] Jacobtorweihe L et al (2025) High cell density perfusion process of quail cells producing oncolytic rVSV-NDV. Eng Life Sci 25(7):e70035 40661158 10.1002/elsc.70035 PMC12256980

[CR23] Jug H, Hosta N,Tajnik M, Strancar A (2013) Production and purification of Newcastle disease virus. BioProcess International

[CR24] Kamen A, Henry O (2004) Development and optimization of an adenovirus production process. J Gene Med 6(Suppl 1):S184–S192 14978761 10.1002/jgm.503

[CR25] Lawler SE et al (2017) Oncolytic viruses in cancer treatment: a review. JAMA Oncol 3(6):841–849 27441411 10.1001/jamaoncol.2016.2064

[CR26] Lee GM et al (1998) Temperature significantly affects retroviral vector production and deactivation rates, and thereby determines retroviral titers. Bioprocess Eng. 10.1007/s004490050530

[CR27] Leon A et al (2016) The EB66(R) cell line as a valuable cell substrate for MVA-based vaccines production. Vaccine 34(48):5878–5885 27997338 10.1016/j.vaccine.2016.10.043

[CR28] Li X et al (2021) Mutations in the methyltransferase motifs of L protein attenuate Newcastle disease virus by regulating viral translation and cell-to-cell spread. Microbiol Spectr 9(2):e0131221 34585949 10.1128/Spectrum.01312-21 PMC8557825

[CR29] Liu T et al (2019) Hemagglutinin-neuraminidase and fusion genes are determinants of NDV thermostability. Vet Microbiol 228:53–60 30593380 10.1016/j.vetmic.2018.11.013

[CR30] Lohr V et al (2012) Live attenuated influenza viruses produced in a suspension process with avian AGE1.CR.pIX cells. BMC Biotechnol 12:79 23110398 10.1186/1472-6750-12-79 PMC3505166

[CR31] Nestler L, Evege E, McLaughlin J, Munroe D, Tan T, Wagner K, Stiles B (2004) TrypLETM Express: a temperature stable replacement for animal trypsin in cell dissociation applications. Quest 1(1):42-47

[CR32] Madeline B, Ribaud S, Xenopoulus A, Simler J, Schamborn K (2015) Culturing a duck ES-derived cell line in single-use bioreactors. BioProcess International 13(3)

[CR33] Nagai Y, Klenk HD, Rott R (1976) Proteolytic cleavage of the viral glycoproteins and its significance for the virulence of Newcastle disease virus. Virology 72(2):494–508 948870 10.1016/0042-6822(76)90178-1

[CR34] Nan FL et al (2021) Lentogenic NDV V protein inhibits IFN responses and represses cell apoptosis. Vet Microbiol 261:109181 34399297 10.1016/j.vetmic.2021.109181

[CR35] Nikolay A (2020) Intensified yellow fever and Zika virus production in animal cell culture. Dissertation; Otto-von-Guericke University Magdeburg

[CR36] Nikolay A (2018) Process intensification of EB66(R) cell cultivations leads to high-yield Yellow fever and Zika virus production. Appl Microbiol Biotechnol 102(20):8725–8737 30091043 10.1007/s00253-018-9275-z PMC6153634

[CR37] Olivier S et al (2010) EB66 cell line, a duck embryonic stem cell-derived substrate for the industrial production of therapeutic monoclonal antibodies with enhanced ADCC activity. MAbs 2(4):405–15 20562528 10.4161/mabs.2.4.12350 PMC3180087

[CR38] Pathak U, Malik N, Pal RB (2022) NDV as an oncolytic agent - study in cancer cell lines. Biosci Biotechnol Res Asia 19(2):413–421

[CR39] Peeters BP et al (1999) Rescue of Newcastle disease virus from cloned cDNA: evidence that cleavability of the fusion protein is a major determinant for virulence. J Virol 73(6):5001–5009 10233962 10.1128/jvi.73.6.5001-5009.1999 PMC112544

[CR40] Pelz L et al (2022) Upstream processing for viral vaccines–general aspects. Bioprocessing of viral vaccines. pp 79–135

[CR41] Reichard KW et al (1992) Newcastle-disease virus selectively kills human tumor-cells. J Surg Res 52(5):448–453 1619912 10.1016/0022-4804(92)90310-v

[CR42] Romer-Oberdorfer A et al (1999) Generation of recombinant lentogenic Newcastle disease virus from cDNA. J Gen Virol 80(Pt 11):2987–2995 10580061 10.1099/0022-1317-80-11-2987

[CR43] Santry LA et al (2018) Production and purification of high-titer Newcastle disease virus for use in preclinical mouse models of cancer. Mol Ther Methods Clin Dev 9:181–191 29556508 10.1016/j.omtm.2017.10.004 PMC5854916

[CR44] Schirrmacher V (2016) Fifty years of clinical application of Newcastle disease virus: time to celebrate! Biomedicines. 10.3390/biomedicines4030016 28536382 10.3390/biomedicines4030016 PMC5344264

[CR45] Schneider M, Marison IW, von Stockar U (1996) The importance of ammonia in mammalian cell culture. J Biotechnol 46(3):161–185 8672289 10.1016/0168-1656(95)00196-4

[CR46] Shalhout SZ et al (2023) Therapy with oncolytic viruses: progress and challenges. Nat Rev Clin Oncol 20(3):160–177 36631681 10.1038/s41571-022-00719-w

[CR47] Shen CF et al (2019) Development of suspension adapted Vero cell culture process technology for production of viral vaccines. Vaccine 37(47):6996–7002 31288997 10.1016/j.vaccine.2019.07.003

[CR48] Sinkovics J (1957) Studies on the biological characteristics of the Newcastle disease virus (NDV) adapted to the brain of newborne mice. Arch Gesamte Virusforsch 7(4):403–411 13521956 10.1007/BF01240748

[CR49] Sinkovics JG, Horvath JC (2000) Newcastle disease virus (NDV): brief history of its oncolytic strains. J Clin Virol 16(1):1–15 10680736 10.1016/s1386-6532(99)00072-4

[CR50] Vaha-Koskela MJ, Heikkila JE, Hinkkanen AE (2007) Oncolytic viruses in cancer therapy. Cancer Lett 254(2):178–216 17383089 10.1016/j.canlet.2007.02.002 PMC7126325

[CR51] Valneva (2026) EB66 Cell line. Available from: https://valneva.com/products/eb66/cell/line. Accessed 29 Apr 2026

[CR52] Vazquez-Ramirez D et al (2018) High-cell-density cultivations to increase MVA virus production. Vaccine 36(22):3124–3133 29433897 10.1016/j.vaccine.2017.10.112 PMC7115588

[CR53] Wechuck JB et al (2002) Effect of temperature, medium composition, and cell passage on production of herpes-based viral vectors. Biotechnol Bioeng 79(1):112–119 17590937 10.1002/bit.10310

[CR54] White KM et al (2018) Influenza B virus reverse genetic backbones with improved growth properties in the EB66(R) cell line as basis for vaccine seed virus generation. Vaccine 36(9):1146–1153 29395518 10.1016/j.vaccine.2018.01.050

[CR55] Workman P et al (2010) Guidelines for the welfare and use of animals in cancer research. Br J Cancer 102(11):1555–77 20502460 10.1038/sj.bjc.6605642 PMC2883160

[CR56] Wu Y et al (2021) High cell density perfusion process for high yield of influenza A virus production using MDCK suspension cells. Appl Microbiol Biotechnol 105(4):1421–1434 33515287 10.1007/s00253-020-11050-8 PMC7847233

[CR57] Yang H et al (2024) The application of Newcastle disease virus (NDV): vaccine vectors and tumor therapy. Viruses. 10.3390/v16060886 38932177 10.3390/v16060886 PMC11209082

[CR58] Yurchenko KS et al (2018) Oncolytic effect of wild-type Newcastle disease virus isolates in cancer cell lines in vitro and in vivo on xenograft model. PLoS One 13(4):e0195425 29621357 10.1371/journal.pone.0195425 PMC5886573

[CR59] Zamarin D et al (2017) Intratumoral modulation of the inducible co-stimulator ICOS by recombinant oncolytic virus promotes systemic anti-tumour immunity. Nat Commun 8(1):14340 28194010 10.1038/ncomms14340 PMC5316835

[CR60] Zinnecker T, Reichl U, Genzel Y (2024) Innovations in cell culture-based influenza vaccine manufacturing - from static cultures to high cell density cultivations. Hum Vaccin Immunother 20(1):2373521 39007904 10.1080/21645515.2024.2373521 PMC11253887

[CR61] Zinnecker T et al (2025) Seed train intensification and TFDF-based perfusion for MDCK cell-based influenza A virus production. Processes. 10.3390/pr13051286

